# Regeneration in Chronic Dento-Alveolar Abscess

**Published:** 1920-08

**Authors:** Julio Endelman


### REGENERATION IN CHRONIC DENTO-ALVEOLAR ABSCESS

BY JULIO ENDELMAN, D.D.S.

The presence of bone involvement beyond and around the apical area is determined radiographically by the decreased density in the calcified tissues; the lesion develops by continuity from the infection in the root canal and peridental membrane and is a typical chronic infectious osteomyelitis. While it may matter nothing from the standpoint of bacteria and bacteria toxin absorption as to how small or how large the rarified area may be, inasmuch as the smallest possible area may in time produce systemic involvements of the severest possible type, from the standpoint of diagnosis and treatment, the extent of involvement presents data of considerable diagnostic significance. It is a fundamental fact in the study of tissue regeneration that if the vitality of the cells of a tissue are not permanently impaired by bacterial, chemical or mechanical irritation and if the source of such irritation be eliminated, processes of repair will take place immediately thereafter and the obliteration of the destroyed area will take place—the tissue destroyed will be replaced by new tissue from proliferations of the fixed cells surrounding the area. This is as true in the case of calcified tissue as in the case of soft tissue. The prevailing views concerning the physiology and pathology of bone are confusing, especially in regard to the possibilities of bone regeneration in connection with chronic dento-alveolar abscesses upon the roots of teeth. If all bacteria have been destroyed in the root canal and periapical region and a root canal filling inserted which reaches to the apex of the root, in certain selected cases bone regeneration will take place with complete disappearance of the area of rarefaction. The cases adapted for treatment are not difficult to diagnose, although before making suggestions along that line, let us bear in mind the following points: *First,* the larger the area to be obliterated by regenerated tissue, the less become the probabilities of a successful termination of the treatment; *second,* a large area may involve less extent of periapical peridental membrane than a small area; *third,* total destruction of periapical peridental membrane does not occur in all cases as may be shown by means of microscopical preparations; *fourth,* the absence of areas of rarefaction does not rule out bacteria and bacterial toxin absorption from the root canals; *fifth,* one radiograph alone is insufficient in the diagnosis of the average chronic dento-alveolar abscess.

Some months ago we brought out editorially pathologic observations concerning microscopic preparations of certain chronic dento-alveolar abscesses. We considered these findings of sufficient importance to publish them without making as many observations as would be desirable. Nevertheless, one of the conclusions reached from those observations was that the peridental membrane in the root area involved by a chronic dento-alveolar abscess is not totally destroyed in every case. That bony regeneration occurs in some instances following the sterilization and filling of root canals is an indisputable fact and as far as the root end is concerned such a phenomenon is possible in the event of either one of two conditions occurring, to-wit: The cementum in the involved area continues to receive nourishment through the fibers remaining attached to it or a regeneration of peridental membrane takes place. Bone regeneration is unthinkable in the presence of a lifeless, inert, cementum and it is our belief that when it does occur the destruction of peridental fibers has been only partial, the alveolar ends only being liquified or destroyed. The cemental ends remain attached to the apical cementum and provide nourishment to the cementum by means of the vessels which penetrate the peridental membrane from the alveolar walls and from the gingiva. Teeth with areas of rarefaction extending vertically rather than laterally and vertically not more than one millimeter as the maximum should be considered favorable cases from the standpoint of eventual obliteration of the area of rarefaction. The greater the lateral involvement the greater must of necessity be the extent of peridental membrane which has suffered by the inroads of the infection. Bone will regenerate regardless of the presence or absence of periosteum; the functions of periosteum have been greatly exaggerated in the past, inasmuch as extensive sections of bone may be replaced by calcified tissue generated through the agency of the myelitic substance contained in the cancellated spaces. While the peridental membrane performs periosteal functions to the internal layers of the alveolar process, areas of bone in the jaws are regenerated by the tissue content of the cancellated spaces. With cementum, conditions differ inasmuch as this tissue has no vascular or nervous supply of its own, depending entirely upon the vitality of the peridental membrane for its existence. In the absence of a portion of the peridental membrane, that area of cementum normally covered by it will degenerate and eventually undergo necrosis. But, as previously remarked a chronic dento-alveolar abscess does not necessarily imply the destruction in toto of the peridental membrane and small areas of peridental tissue in the absence of infection to regenerate.—*Pacific Dental Gazette.*

